# Physical activity when riding an electric assisted bicycle

**DOI:** 10.1186/s12966-017-0513-z

**Published:** 2017-04-26

**Authors:** Sveinung Berntsen, Lena Malnes, Aleksander Langåker, Elling Bere

**Affiliations:** Deparment of Public Health, Sport and Nutrition, University of Agder, Post Box 422, NO-4604 Kristiansand, Norway

**Keywords:** Electrically assisted bicycle, Commuting, Intensity, Oxygen uptake, Pedalecs

## Abstract

**Background:**

The objectives of the present study were to compare time spent cycling, exercise intensity, and time spent in moderate- (MPA) and vigorous intensity physical activity (VPA) when cycling on an E-bike and a conventional bicycle on two “cycling-to-work” routes with differences in topography, defined as a hilly and a flat route.

**Methods:**

Eight adults (23–54 years, two women) cycled outdoors on a conventional bicycle and an E-bike, on a flat (8.2 km) and a hilly (7.1 km) route, resulting in 32 journeys. Duration, elevation, and oxygen consumption were recorded using a portable oxygen analyser with GPS. A maximal cardiorespiratory fitness test was performed on a cycle ergometer. Resting metabolic rate was obtained by indirect calorimetry with a canopy hood.

**Results:**

The participants spent less time (median (IQR)) cycling on the E-bike compared with the conventional bicycle, on both the hilly (18.8 (4.9) vs. 26.3 (6.4) minutes) and the flat (20.0 (2.9) vs. 23.8 (1.8) minutes) routes. Lower exercise intensity was observed with the E-bike compared with the conventional bicycle, both on the hilly (50 (18) vs. 60 (22) % of maximal oxygen uptake) and the flat (52 (19) vs. 55 (12) % of maximal oxygen uptake) routes. In both cycling modes, most time was spent in MVPA (92–99%). However, fewer minutes were spent in MVPA with the E-bike than the conventional bicycle, for both the hilly (26% lower) and the flat (17% lower) routes. Cycling on the E-bike also resulted in 35 and 15% fewer minutes in vigorous intensity, respectively on the hilly and flat routes.

**Conclusion:**

Cycling on the E-bike resulted in lower trip duration and exercise intensity, compared with the conventional bicycle. However, most of the time was spent in MVPA. This suggests that changing the commuting mode from car to E-bike will significantly increase levels of physical activity while commuting.

## Background

Physical activity can have a beneficial effect on health and fitness [1, 2]. Unfortunately, most adults are insufficiently active [3], below the recommended 150 min physical activity of moderate intensity or 75 min of vigorous intensity per week [4].

In the last decades, research has indicated an increase in exercise training during leisure time [5], and a reduction in household- [6], work-, and transport-related physical activity [5]. Promotion of transport-related physical activity has traditionally focused on walking and cycling [7]. Meanwhile, electric assisted bicycles (E-bikes) have become increasingly popular [8]. E-bike users have reported advantages such as higher speed with less effort, reduced travel time and easier to climb hills compared to conventional bicycles [9, 10]. In Europe, E-bikes provide electrical assistance only when the bike rider is pedalling [11], thus is partly human powered. Moreover, E-bikes seem to be highly used for commuting purposes [10, 12–14]; therefore, it is essential to establish whether cycling with an E-bike can be health-enhancing, which depends on duration and intensity of physical activity [15].

Intensity can be measured as relative, such as percentage of maximal oxygen uptake ($\overset{.}{\mathrm{V}}{\mathrm{O}}_2 \max$) or absolute as metabolic equivalent of tasks (METs), where one MET is defined as resting metabolic rate (RMR). However, without direct measurements, RMR is usually replaced by a 1-MET reference value of 3.5 ml O_2_/kg/min [16]. Intensity when riding a conventional bicycle has been classified as vigorous, ranging from 6.4 to 8.2 METs [16–19]. Recent studies on E-bikes have reported somewhat lower intensity, ranging from 4.1 to 6.1 METs [19–22]. Some studies [20–22] have compared cycling with no- and maximal electrical power; however, an E-bike is heavier than a conventional bicycle and therefore E-biking with the power switched off does not fairly represent cycling on a conventional bicycle [8, 23].

Gojanovic et. al.[19] investigated direct comparisons between cycling on an E-bike and a conventional bicycle. They found lower exercise intensity, measured as percentage of $\overset{.}{\mathrm{V}}{\mathrm{O}}_2 \max$, on an E-bike (55%) compared with a conventional bicycle (73%) on an uphill route. However, the lower cycling intensity is likely to vary according to topography. In addition, previous studies [19–22] have estimated METs based on the standard 1-MET of 3.5 ml O_2_/kg/min. Since RMR may be influenced by age, gender, fat-free mass, body mass index (BMI) and fitness level [24, 25], direct measurements are preferred.

The objectives of the present study were to compare time spent cycling, exercise intensity, and time spent in moderate- (MPA) and vigorous intensity physical activity (VPA) when cycling on an E-bike and a conventional bicycle on two “cycling-to-work” routes with differences in topography, defined as a hilly and a flat route.

## Subjects and Methods

### Participants and study design

Six men and two women (23–54 years of age) met in the morning (07.00–10.00 am) for measurements of RMR. Thereafter, each participant performed four field-cycling tests outdoors and a maximal cardiorespiratory fitness test on a cycle ergometer in the laboratory. All participants were of Caucasian origin, non-smokers and without overt disease or use of medications.

The participants were given oral and written information about the study objectives and methods. Data was stored according to guidelines by the Norwegian Social Science Data Services.

### Measurements

Height and body mass were measured (in light clothing, without shoes) using a stadiometer and a physician’s scale (Seca 713, Birmingham, UK).

#### Field tests

Oxygen uptake ($\overset{.}{\mathrm{V}}{\mathrm{O}}_2$) was measured with a portable oxygen analyser (MetaMax 3B-R2, CORTEX Biophysik GmbH, Leipzig, Germany) and a breathing mask, which were dressed according to the instructions of the manufacturer. MetaMax 3B-R2 has been found reliable and valid (approximately 10% difference between methods) when compared to the “Gold standard” the Douglas bag method and a secondary criterion machine known to be accurate, the Jaeger Oxycon Pro system [26]. Prior to using, the system was turned on for at least 30 min, and then calibrated prior to every test. First indoor calibrating of the gas analysers by using a reference gas (16% O_2_, 4% CO_2_), and then verifying the calibration against ambient air. Secondly, a volume calibration was performed using a standardised 3-L syringe (5530 series, Hans Rudolph, Inc., MO, USA). Sensor adjustments (ambient air calibration) were performed after bringing the MetaMax outside and before the first cycling session in each person. Speed and elevation were measured from GPS-coordinates using a GPS Kit for MetaMax® and cycling time was measured on-site by the test leader.

Total measurement period lasted between 120 and 150 min, where the participants cycled on two “cycling-to-work” routes, using a conventional bicycle (Cannondale 50/50 with an internal Shimano Nexus 8 speed hub, wheel size 26 `and bike weight of 12 kg (2005 model), Wilton, Connecticut, USA) and an E-bike cycling on maximal electrical power (RIXE with a 17 Ah/612 Wh Impulse battery, wheel size 28 `and bike weight of 27 kg (2014 model), Cloppenburg, Germany). The E-bike had a motor capacity of 250 W and a maximal speed of 25 km/h, with an active engine.

**Fig. 1:**
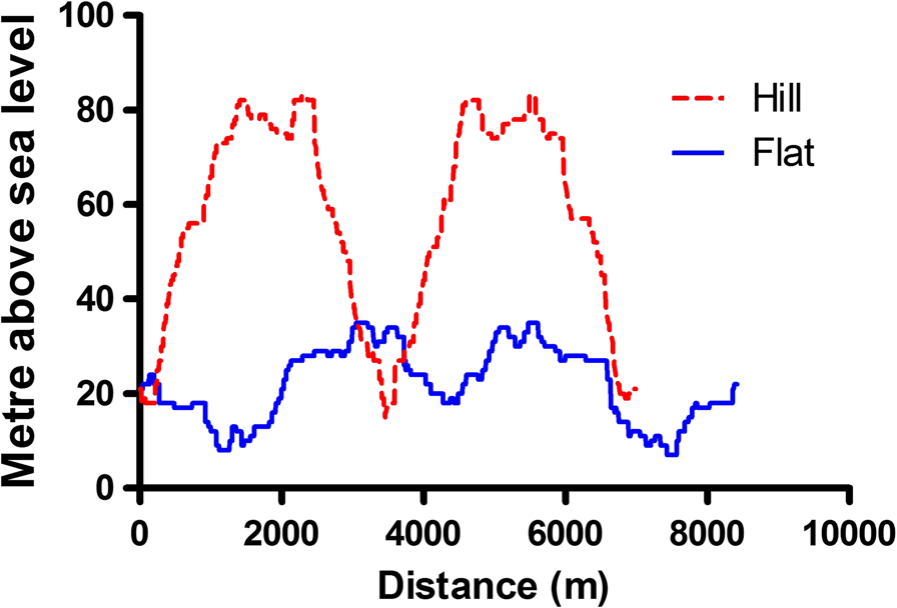
The elevation profile for the flat (*solid*) and the hilly (*dotted*) course

The two routes (a flat route of 8.1 km, and a hillier, but shorter, route of 7.1 km) started at the same place of departure, reached the same destination, and ended at the place of departure (simulating commuting, back and forth, from residence to a place of work). Altitude at both departure and destination was 18 m above sea level. The hilly route reached a maximal altitude of 83 m above sea level, including one hill that was climbed twice (to and from destination giving a total height difference of 130 m). Conversely, the flat route had no steep hills, and reached a maximal altitude of 35 m above sea level (total height difference 34 m). The route profiles are shown in Fig. 1.

Each participant performed four trips, the order of which was randomised; these were 1) E-bike hilly, 2) E-bike flat, 3) Bike hilly and 4) Bike flat. There was a two minutes’ break between the experimental conditions. The participants were told to cycle at the intensity they would choose when commuting to work.

#### Laboratory tests

Before the field tests, RMR was obtained by indirect calorimetry with a canopy hood (Oxycon Pro, Jaeger BeNeLux Bv, Breda, Netherlands) according to international guidelines [27], using a standardised protocol. Prior to using, the system was turned on for at least 30 min, and then calibrated prior to every test. First calibrating of the gas analysers by using a reference gas (15% O_2_, 6% CO_2_), and then verifying the calibration against ambient air. Secondly, an automatic volume calibration was performed according to manufacturer’s recommendations. Participants were instructed to abstain from coffee, alcohol, smoking, exercise and not eat food, 12 h before measurements. The participants were placed under the canopy hood in a relaxed, supine position for 30 min and instructed to stay awake. Measurements were repeated on all participants until a 15-min steady state and the lowest $\overset{.}{\mathrm{V}}{\mathrm{O}}_2$ (ml/kg/min) value, average of one minute, defined RMR.

After the field tests, the participants had a 15 min’ break before they performed a test of cardiorespiratory fitness, measured as $\overset{.}{\mathrm{V}}{\mathrm{O}}_2 \max$. The protocol was performed using an electronically-braked cycle ergometer (Monark 839 Ergomedic, Varberg, Sweden), which was calibrated electronically before each test and mechanically after being moved. MetaMax 3B-R2 was calibrated before each test. During $\overset{.}{\mathrm{V}}{\mathrm{O}}_2 \max$ tests, the ambient air temperature was 22 – 23 °C, relative humidity 50 – 60%, and barometric pressure 99.3 – 101.2 kPa. The initial workload was 50 W and was increased by 25 W every 2nd minutes until exhaustion. The pedalling rate (between 60 and 80 rpm) was instructed, and height of the saddle was adjusted individually. Minute ventilation ($\overset{.}{\mathrm{V}}\mathrm{E}$), respiratory exchange ratio (RER) and $\overset{.}{\mathrm{V}}{\mathrm{O}}_2$ were recorded every minute by using the same portable gas analyser as in the field tests. Data was analysed with Metasoft Studio v.4.9 (Cortex Biophysik, Leipzig, Germany). The main criterion for having reached maximal effort was a subjective assessment by the test leader. The second criteria were RER above 1.00 and reporting perceived exertion (RPE) above 17 using the Borg-RPE-Scale [28].

### Data processing

Data from start to end of cycling the sessions were imported into Microsoft Excel®, computed at one-minute intervals, and synchronised for further analysis. Percentage of $\overset{.}{\mathrm{V}}{\mathrm{O}}_2$ max was calculated by dividing $\overset{.}{\mathrm{V}}{\mathrm{O}}_2$ (ml/kg/min) from the field tests by maximal $\overset{.}{\mathrm{V}}{\mathrm{O}}_2$ (ml/kg/min) from the laboratory test. Measured METs and estimated METs were calculated by dividing participants RMR ($\overset{.}{\mathrm{V}}{\mathrm{O}}_2$ ml/kg/min) and the standard 1-MET value (3.5 ml O_2_/kg/min), respectively, by oxygen uptake during the field tests. Time spent in moderate and vigorous intensity physical activity was based on measured METs, which was categorised as moderate if 3 – 5.9 METs or vigorous if ≥6 METs [29]. Data are presented as median and interquartile range (IQR).

## Results

Baseline characteristics of the participants are shown in Table 1. Results from a total of 32 trips are presented.

**Table 1:** Physical characteristics of the eight participating subjects (median, interquartile range (IQR), minimum (Min) and maximum (Max)

	Median	IQR	Min-Max
Age (yrs)	39	13	23–54
Body mass (kg)	74	13	64–86
Height (cm)	177	7	169–184
BMI (kg⋅m^-2^)	24	5	21–27
RMR (ml O_2_ ⋅ kg ^-1^ ⋅ min^-1^)	3.0	0.2	2.6–3.8
HR rest (beats⋅ min^-1^)	45	6	34–49
$\overset{.}{\mathrm{V}}{\mathrm{O}}_2 \max$ (ml · kg ^-1^ · min^-1^)	45.0	17.4	36.3–81.8
HRmax (beats⋅ min^-1^)	177	9	169–185

*Abbreviations*: *BMI* Body Mass Index, *RMR* Resting Metabolic Rate, *HRmax* maximal heart rate, $\overset{.}{\mathrm{V}}{\mathrm{O}}_2 \max$ maximal oxygen uptake

### Time spent cycling

**Fig. 2:**
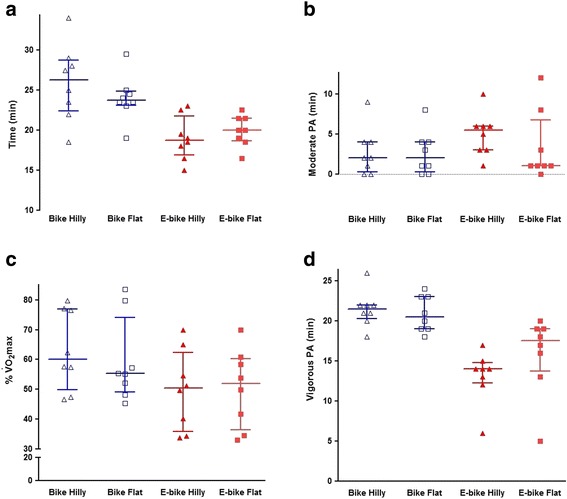
Time spent cycling (**a**), percentage of VO_2_max (%VO_2_max) (**c**) and time spent in moderate (**b**) and vigorous (**d**) intensity physical activity per 10 km during flat (empty) and hilly (filled) on the E-bike (quadrate) and the regular bicycle (triangle). Presented as median and interquartile range

The median cycling time for the two routes combined was 19.9 min for E-bike and 25.1 min for conventional bicycle, i.e. the E-bike was 21% faster (Table 2 and Fig. 2). Median speed was 23.1 km/h for E-bike and 18.4 km/h for conventional bicycle, a difference of 4.7 km/h. The difference between E-bike and conventional bicycle was greater on the hilly route (E-bike 29% faster) than on the flat route (E-bike 16% faster).

**Table 2:** Median (interquartile range) and percentage differences in cycling time, speed, exercise intensity (% of maximal oxygen consumption), energy expenditure (metabolic equivalents attained during cycling based on 1 MET–3.5 ml O_2_ · kg ^-1^ · min^-1^ and measured RMR), moderate-to-vigorous intensity physical activity and vigorous intensity physical activity during electrically assisted- and conventional biking presented combined and stratified on flat and hilly

	Total	Flat	Hilly
Cycling time (min)			
E-bike	19.9 (3.1)	20.0 (2.9)	18.8 (4.9)
Conventional	25.1 (3.9)	23.8 (1.8)	26.3 (6.4)
Δ (%)	-21	-16	-29
Speed (km · h^-1^)			
E-bike	23.1 (3.7)	24.6 (3.5)	22.7 (5.6)
Conventional	18.4 (2.7)	20.7 (1.5)	16.3 (4.2)
Δ (%)	21	16	29
% $\overset{.}{\mathrm{V}}{\mathrm{O}}_2 \max$			
E-bike	51 (27)	52 (19)	50 (18)
Conventional	58 (26)	55 (12)	60 (22)
Δ (%)	-12	-6	-17
Measured METs (ml · kg ^-1^ · min^-1^)			
E-bike	8.5 (3.1)	8.4 (3.3)	8.4 (3.2)
Conventional	10.9 (2.7)	10.3 (2.8)	10.8 (3.1)
Δ (%)	-22	-19	-22
Estimated METs (ml · kg ^-1^ · min^-1^)			
E-bike	6.9 (2.1)	6.9 (1.9)	6.8 (2.5)
Conventional	8.4 (1.8)	8.1 (2.5)	8.5 (2.1)
Δ (%)	-18	-15	-20
MVPA (min)			
E-bike	19.0 (2.7)	19.5 (3.4)	18.0 (2.8)
Conventional	23.9 (3.6)	23.5 (2.6)	24.3 (4.6)
Δ (%)	-21	-17	-26
VPA (min)			
E-bike	15.5 (4.1)	17.5 (5.3)	14.0 (2.5)
Conventional	20.8 (3.1)	20.5 (4.0)	21.5 (1.8)
Δ (%)	-26	-15	-35

*Abbreviations*: *E-bike* electrically assisted bicycle, *Δ* percentage difference between electrically assisted- and conventional biking; $\overset{.}{\mathrm{V}}{\mathrm{O}}_2$ oxygen consumption, *METs* Metabolic Equivalents, *MVPA* moderate-to-vigorous intensity physical activity, *VPA* vigorous intensity physical activity

### Exercise intensity

Exercise intensity presented as $\overset{.}{\mathrm{V}}{\mathrm{O}}_2 \max$ for the E-bike and the conventional bicycle was for the routes combined 51 and 58%, respectively (a difference of 12%). The difference between the bikes was greater on the hilly route than on the flat route, 17 vs 6%, respectively. Expressed as METs, the exercise intensity over the two routes using an E-bike was measured to be 8.5 METs, while using conventional bicycle was measured to be 10.9. Using METs from the estimated resting metabolic rate of 3.5 ml O_2_/kg/min, the estimated METs were respectively 6.9 and 8.4.

### Time spent in moderate- and vigorous intensity physical activity

In total, 19.0 and 23.9 min were spent in MVPA when using measured METs, respectively for E-bike and conventional bicycle, which was 95% of total cycling time for both cycle types. Most of the time in both the hilly and flat routes were spent in MVPA, however, due to less time spent cycling on the hilly route, the time in MVPA (26% lower), and especially in VPA (35% lower) was lower using E-bike compared to conventional bicycle.

## Discussion

In the present study, comparing e-biking to conventional bicycling on two different routes simulating relevant cycling to work options, e-biking was faster and less intensive than conventional bicycling, especially on the hilly route. However, 95% of time spent biking, both for e-bike and conventional bike, were considered to be MVPA.

E-biking were faster than conventional bicycling, and reduced the time cycling with almost 30% in the hilly route. These results support the findings from previous studies regarding lower trip duration with an E-bike compared with a conventional bicycle [19, 30, 31], due to a higher speed. Gojanovic et al. [19] observed that when riding an E-bike, speed was on average 6 km/h higher than on a conventional bicycle, on the same route, similar to the 5 km/t in the present study. Furthermore, observational studies found on average 2 km/h higher speed in E-bike cyclists compared with individuals travelling by conventional bicycles [30, 31]. Schleinitz et al. [30] also illustrated that age, road gradient, and bicycle infrastructure may influence differences in cycling speed and trip duration between E-bikes and conventional bicycles.

E-biking was less intense than conventional bicycling, both relative (a 17% lower $\overset{.}{\mathrm{V}}{\mathrm{O}}_2 \max$) and absolute (36% lower time spent in VPA). To the authors´ knowledge, in only one published study exercise intensity has been measured relative to cardiorespiratory fitness when riding an E-bike and a conventional bicycle. Gojanovic et al. [19] observed 55% of $\overset{.}{\mathrm{V}}{\mathrm{O}}_2 \max$ when subjects cycled on an E-bike, and 72% of $\overset{.}{\mathrm{V}}{\mathrm{O}}_2 \max$ during conventional bicycling, and slightly higher compared to the 51 and 58% in present study. In the present study, when the participants cycled on the conventional bicycle, the difference between the hilly and flat route was larger, probably due to higher physical effort cycling uphill without electrical support. Therefore, the findings may suggest that conventional bicycles are more sensitive to individual- and environmental factors, such as topography. Compared to studies on conventional bicycles, Oja and colleagues [32] supported the present findings, in 68 commuting adults, as the relative intensity was 57–65% of $\overset{.}{\mathrm{V}}{\mathrm{O}}_2 \max$, whereas Geus et al. [17] observed a higher intensity of 77–79% of $\overset{.}{\mathrm{V}}{\mathrm{O}}_2 \max$.

Exercise intensity, presented as METs, was in the present study also lower when subjects cycled on the E-bike compared to the conventional bike, however, figures for the hilly and flat routes were similar. The explanation for similar average MET-values during the flat and hilly routes are the periods of downhill with lower energy demand following the periods of ascending during hilly biking. A few previous studies have presented MET-values (4–6 METs) for E-bikes when cycling on a varied terrain [19–22]. These findings were similar, or somewhat lower, to the estimated METs in the present study, however, considerable lower than the measured METs. Presenting energy expenditure or exercise intensity using the standard 1-MET value (3.5 ml O_2_/kg/min) may result in lower reported levels in individuals since the standard 1-MET value has been reported to be overestimating resting metabolic rate by 35 and 14% in individuals with a mean BMI of 30 and 20 kg/m^2^, [25], respectively, and similar to the present study (resting metabolic rate of 3.0 ml O_2_/kg/min). However, our results indicate that both using the standard 1-MET or measured RMR, e-biking can be categorised as at least MVPA.

E-biking was faster and less intense, making it suited for busy modern lives. It will get you quicker to work and you might not need a shower. But, on the other hand, less time spent cycling at a lower intensity is not ideal as most people are inactive. However, most of the time spent cycling on both the E-bike and the conventional bicycle, in both routes, was spent in MVPA.

The present findings indicate that due to lower intensity and trip duration, an E-bike needs to be used more frequently or to cover longer distances, to achieve the same health benefits [15], as adults commuting by a conventional bicycle. On the other hand, in several European nations, most adults do commute by motorised transportation to work [33], which according to Costa et al. [18] can be categorised as sedentary behaviour or light intensity physical activity (below 3 METs). Since the present findings indicate at least a moderate intensity of physical activity when e-biking, an increase in the level of transport-related physical activity will follow switching from car commuting to E-bikes, and more individuals probably meeting the physical activity recommendations. Also, considering that frequently reported barriers to cycling or walking for transport have been travel time [34–37] and physical effort [36, 37]; the E-bike seems to be a good option for those living too far away from work to walk or cycle with a conventional bike.

### Strength and limitations

The main strengths of the present study were the objective measurements of RMR, direct measurements of $\overset{.}{\mathrm{V}}{\mathrm{O}}_2 \max$ in the laboratory and $\overset{.}{\mathrm{V}}{\mathrm{O}}_2$ during the field tests as well as using GPS to measure elevation and distance covered. However, due to a small sample size not the findings are presented as descriptive rather than analytical. During the field tests, the participants may have cycled at a higher intensity, due to participating in an experimental study. In addition, the cycling trips is not necessarily representative to all cycling trips in Norway. The participants performed the four field tests on the same day, which may have affected our results. However, the route and type of bicycle were selected in random order.

### Conclusion

Cycling on the E-bike resulted in lower trip duration and exercise intensity, compared with the regular bicycle. However, most of the time was spent in MVPA. Therefore, changing commuting mode from car to E-bike will significantly increase levels of physical activity while commuting.

## Abbreviations

$\overset{.}{\mathrm{V}}{\mathrm{O}}_2 \max$: maximal oxygen uptake
$$ \overset{.}{\mathrm{V}}\mathrm{E} $$: minute ventilation
$$ \overset{.}{\mathrm{V}}{\mathrm{O}}_2 $$: oxygen uptake
BMI: Body mass index
E-bike: Electrical assisted bicycle
IQR: Inter quartile range
Km: Kilometres
km/h: Kilometres per hour
kPa: Kilopascal
METs: Metabolic equivalents
Min: Minutes
MVPA: Moderate-to-vigorous intensity physical activity
RER: Respiratory exchange ratio
RMR: Resting metabolic rate
RPE: Reporting perceived exertion
Rpm: Revolutions per minutes
W: Watt

## References

[1] Lee I.-M, Shiroma EJ, Lobelo F, Puska P, Blair SN, Katzmarzyk PT. Effect of physical inactivity on major non-communicable diseases worldwide: an analysis of burden of disease and life expectancy. The Lancet. 2012;380(9838). doi: http://dx.doi.org/10.1016/s0140-6736(12)61031-9. 10.1016/S0140-6736(12)61031-9 PMC3645500 22818936

[2] Kumanyika SK, Obarzanek E, Stettler N, Bell R, Field AE, Fortmann SP, et al. Population-based prevention of obesity: the need for comprehensive promotion of healthful eating, physical activity, and energy balance: a scientific statement from American Heart Association Council on Epidemiology and Prevention, Interdisciplinary Committee for Prevention (formerly the expert panel on population and prevention science). Circulation. 2008;118(4). doi: http://dx.doi.org/10.1161/circulationaha.108.189702. 10.1161/CIRCULATIONAHA.108.189702 18591433

[3] Hallal PC, Andersen LB, Bull FC, Haskell GRW, Ekelund U. Global physical activity levels: surveillance progress, pitfalls, and prospects. The Lancet. 2012;380(9838). doi: http://dx.doi.org/10.1016/s0140-6736(12)60646-1. 10.1016/S0140-6736(12)60646-1 22818937

[4] World Health Organisation. Global recommendations on physical activity for health. 2010 [cited 2015 31.mai]; Available from: http://www.who.int/dietphysicalactivity/publications/9789241599979/en/. 26180873

[5] Borodulin K, Harald K, Jousilahti P, Laatikainen T, M?nnist S, Vartiainen E, et al. Time trends in physical activity from 1982 to 2012 in Finland. Scand J Med Sci Sports. 2015. doi: http://dx.doi.org/10.1016/s0140-6736(12)60646-1. 10.1111/sms.12401 25559167

[6] Archer E, Shook RP, Thomas DM, Church TS, Katzmarzyk PT, Hébert JR, et al. 45-Year Trends in Women’s Use of Time and Household Management Energy Expenditure. PLoS ONE. 2013;8(2). doi: http://dx.doi.org/10.1371/journal.pone.0056620. 10.1371/journal.pone.0056620 PMC3577846 23437187

[7] Saunders LE, Green JM, Petticrew MP, Steinbach R, Roberts H. What are the health benefits of active travel?. A systematic review of trials and cohort studies. PLoS One. 2013;8(8). doi: http://dx.doi.org/10.1371/journal.pone.0069912. 10.1371/journal.pone.0069912 PMC3744525 23967064

[8] Fishman E, Cherry C. E-bikes in the Mainstream: Reviewing a Decade of Research. Transport Reviews. 2015. doi: http://dx.doi.org/10.1080/01441647.2015.1069907.

[9] Langford B, Cherry C, Yoon T, Worley S, Smith D. North America’s First E-Bikeshare A Year of Experience. Transportation Research Record. 2013(2387). doi: http://dx.doi.org/10.3141/2387-14.

[10] Dill J, Rose G. Electric Bikes and Transportation Policy. Transportation Research Record: Journal of the Transportation Research Board. 2012. 2314. doi: http://dx.doi.org/10.3141/2314-01.

[11] European Commision. European Committee for Standardization EN 15194. 2009 [cited 2016 30. Januar]; Available from: https://ec.europa.eu/energy/intelligent/projects/sites/iee-projects/files/projects/documents/presto_fact_sheet_legislation_en.pdf.

[12] Fyhri A, Fearnley N. Effects of e-bikes on bicycle use and mode share. Transportation Research Part D. 2015;36(45). doi: http://dx.doi.org/10.1016/j.trd.2015.02.005.

[13] Popovich N, Gordon E, Shao Z, Xing Y, Wang Y, Handy S. Experiences of electric bicycle users in the Sacramento, California area. Travel Behaviour and Society. 2014;1(2). doi: http://dx.doi.org/10.1016/j.tbs.2013.10.006.

[14] Astegiano P, Tampère CMJ, Beckx C. A Preliminary Analysis Over the Factors Related with the Possession of an Electric Bike. Transportation Research Procedia. 2015;10. doi: http://dx.doi.org/10.1016/j.trpro.2015.09.089.

[15] Haskell WL, Lee I-M, Pate RR, Powell KE, Blair SN, Franklin BA, et al. Physical activity and public health: updated recommendation for adults from the American College of Sports Medicine and the American Heart Association. Circulation. 2007;116(9). doi: http://dx.doi.org/10.1161/circulationaha.107.185649. 10.1161/CIRCULATIONAHA.107.185649 17671237

[16] Ainsworth BE, Haskell WL, Whitt MC, Irwin ML, Swartz AM, Strath SJ, et al. Compendium of physical activities: an update of activity codes and MET intensities. Med Sci Sports Exerc. 2000;32(9; SUPP/1). doi: http://dx.doi.org/10.1097/00005768-200009001-00009. 10.1097/00005768-200009001-00009 10993420

[17] de Geus B, De Smet S, Nijs J, Meeusen R. Determining the intensity and energy expenditure during commuter cycling. Br J Sports Med. 2007;41(1). doi: http://dx.doi.org/10.1136/bjsm.2006.027615. 10.1136/bjsm.2006.027615 PMC2465142 17021003

[18] Costa, S, Ogilvie D, Dalton A, Westgate K, Brage S, Panter J. Quantifying the physical activity energy expenditure of commuters using a combination of global positioning system and combined heart rate and movement sensors. Prev Med. 2015;81. doi: http://dx.doi.org/10.1016/j.ypmed.2015.09.022. 10.1016/j.ypmed.2015.09.022 PMC4678256 26441297

[19] Gojanovic B, Welker J, Iglesias K, Daucourt C, Gremion G. Electric bicycles as a new active transportation modality to promote health. Med Sci Sports Exerc. 2011;43(11). doi: http://dx.doi.org/10.1249/mss.0b013e31821cbdc8. 10.1249/MSS.0b013e31821cbdc8 22005715

[20] Sperlich B, Zinner C, Hebert-Losier K, Born D-P, Holmberg H-C. Biomechanical, cardiorespiratory, metabolic and perceived responses to electrically assisted cycling. Eur J Appl Physiol. 2012;112(12). doi: http://dx.doi.org/10.1007/s00421-012-2382-0. 10.1007/s00421-012-2382-0 22446956

[21] Louis, J, Brisswalter J, Morio C, Barla C, Temprado J-J. The electrically assisted bicycle: an alternative way to promote physical activity. Am J Phys Med Rehabil. 2012;91(11). doi: http://dx.doi.org/10.1097/phm.0b013e318269d9bb. 10.1097/PHM.0b013e318269d9bb 23085705

[22] Simons M, Van Es E, Hendriksen I. Electrically assisted cycling: a new mode for meeting physical activity guidelines?. Med Sci Sports Exerc. 2009;41(11). doi: http://dx.doi.org/10.1249/mss.0b013e3181a6aaa4. 10.1249/MSS.0b013e3181a6aaa4 19812505

[23] MacArthur J, Kobel N (2015). Regulations of E-bikes in the United States: A policy review. In transportation research board 94th annual meeting.

[24] Kozey S, Lyden K, Staudenmayer J, Freedson P. Errors in MET Estimates of Physical Activities Using 3.5 ml. kg-1 min-1 as the Baseline Oxygen Consumption.. J Phys Act Health. 2010;7(4). doi: http://dx.doi.org/10.1123/jpah.7.4.508. 10.1123/jpah.7.4.508 20683093

[25] Byrne NM, Hills AP, Hunter GR, Weinsier RL, Schutz Y. Metabolic equivalent: one size does not fit all. J Appl Physiol. 2005;99(3) . doi: http://dx.doi.org/10.1152/japplphysiol.00023.2004. 10.1152/japplphysiol.00023.2004 15831804

[26] Macfarlane DJ, Wong P. Validity, reliability and stability of the portable Cortex Metamax 3B gas analysis system.. Eur J Appl Physiol. 2012;112(7). doi: 10.1007/s00421-011-2230-7. 10.1007/s00421-011-2230-7 PMC3371330 22075643

[27] Compher C, Frankenfield D, Keim N, Roth-Yousey L and EAW Group. Best practice methods to apply to measurement of resting metabolic rate in adults: a systematic review. J Am Diet Assoc. 2006;106(6). doi: http://dx.doi.org/10.1016/j.jada.2006.02.009. 10.1016/j.jada.2006.02.009 16720129

[28] Borg, G. Perceived exertion as an indicator of somatic stress. Scandinavian journal of rehabilitation medicine. 1970;2(2). 5523831

[29] Garber CE, Blissmer, Deschenes BA, Franklin MJ Lamonte, Lee I-M et al. American College of Sports Medicine position stand. Quantity and quality of exercise for developing and maintaining cardiorespiratory, musculoskeletal, and neuromotor fitness in apparently healthy adults: guidance for prescribing exercise. Med Sci Sports Exerc. 2011;43(7). doi: http://dx.doi.org/10.1249/mss.0b013e318213fefb. 10.1249/MSS.0b013e318213fefb 21694556

[30] Schleinitz K, Petzoldt T, Franke-Bartholdt L, Krems J, Gehlert T. The German Naturalistic Cycling Study – Comparing cycling speed of riders of different e-bikes and conventional bicycles. Safety Science. 2015. doi: http://dx.doi.org/10.1016/j.ssci.2015.07.027

[31] Langford BC, Chen J, Cherry CR. Risky riding: Naturalistic methods comparing safety behavior from conventional bicycle riders and electric bike riders. Accid Anal Prev. 2015;82. doi: http://dx.doi.org/10.1016/j.aap.2015.05.016. 10.1016/j.aap.2015.05.016 26093098

[32] Oja P, Mänttäri A, Heinonen A, Kukkonen-Harjula K, Laukkanen R, Pasanen M, et al. Physiological effects of walking and cycling to work. Scandinavian Journal of Medicine & Science in Sports. 1991;1(3). doi: http://dx.doi.org/10.1111/j.1600-0838.1991.tb00288.x.

[33] Bassett DR Jr, Pucher J, Buehler R, Thompson DL, Crouter SE. Walking, cycling, and obesity rates in Europe, North America, and Australia. J Phys Act Health. 2008;5(6). doi: http://dx.doi.org/10.1123/jpah.5.6.795. 10.1123/jpah.5.6.795 19164816

[34] Bopp M, Kaczynski AT, Besenyi G. Active commuting influences among adults. Preventive Medicine. 2012;54(3–4). doi: http://dx.doi.org/10.1016/j.ypmed.2012.01.016. 10.1016/j.ypmed.2012.01.016 22327047

[35] Kaczynski AT, Bopp MJ, Wittman P. To drive or not to drive: factors differentiating active versus non-active commuters. J Health Behav Public Health. 2012;2(2).

[36] Engbers LH, Hendriksen IJM. Characteristics of a population of commuter cyclists in the Netherlands: perceived barriers and facilitators in the personal, social and physical environment. Int J Behav Nutr Phys Act. 2010;7. doi: http://dx.doi.org/10.1186/1479-5868-7-89. 10.1186/1479-5868-7-89 PMC3012015 21143948

[37] Titze S, Stronegger WJ, Janschitz S, Oja P. Association of built-environment, social-environment and personal factors with bicycling as a mode of transportation among Austrian city dwellers. Prev Med, 2008;47(3). 10.1016/j.ypmed.2008.02.019 18417199

